# Journalistic Notes

**Published:** 1891-07

**Authors:** 


### Journalistic Notes.

A Merited Advancement.—Dr. J. C. Culbertson, the veteran editor of the Cincinnati *Lancet-Clinic*, has accepted the invitation of the trustees to take charge of the editorial and business management of the *Journal of the American Medical Association*, and has already bid adieu to the readers of the *Lancet-Clinic*, and entered upon the discharge of the duties of his new office. We congratulate the trustees upon their wise selection of Dr. Culbertson, which we feel sure will meet the approval of the profession throughout the country, and we congratulate Dr. Culbertson upon his entrance to a larger sphere of work, which will give him greater opportunities for the exhibition of that fine editorial ability which has always characterized his conduct of the *Lancet-Clinic*.

In taking leave of the *Lancet-Clinic*, Dr. Culbertson introduces as his successors Drs. A. B. Richardson, J. C. Oliver and L. S. Colter, of whom he says: "These gentlemen are selected as associate editors, because of their peculiar fitness and ability, and it gives us great pleasure to commend them to our readers as worthy professional gentlemen."

The *Monthly* wishes them a hearty welcome.—*Memphis Medical Monthly.*

---

The *Ophthalmic Record*, of Nashville, Tenn., is most welcome to our exchange list. The first issue, bearing date July, 1891, has already made its appearance, and if it maintains in the future the excellent character of its initial number, it can but succeed. It is under the editorial management of Prof. G. C. Savage, M. D., who is assisted by Dr. Geo. H. Price, who will have charge of the department of otology, laryngology and rhinology. Although, for convenience, bearing as its name, Ophthalmic Record, it will also give full attention to the specialties named above. The first number contains thirty-two pages of carefully prepared literature, well printed on excellent paper. It will be published monthly; subscription price, $2.00 per annum.—*Southern Practitioner.*

